# Activation of STING by the novel liposomal TLC388 enhances the therapeutic response to anti-PD-1 antibodies in combination with radiotherapy

**DOI:** 10.1007/s00262-024-03692-8

**Published:** 2024-04-02

**Authors:** Jhen-Yu Chen, Po-Yu Lin, Wei-Ze Hong, Pei-Chen Yang, Shu-Fen Chiang, Hsin-Yu Chang, Tao-Wei Ke, Ji-An Liang, William Tzu-Liang Chen, K. S. Clifford Chao, Kevin Chih-Yang Huang

**Affiliations:** 1 Department of Biomedical Imaging and Radiological Science, China Medical University, Taichung, 40402 Taiwan; 2 Translation Research Core, China Medical University Hospital, China Medical University, Taichung, 40402 Taiwan; 3 Proton Therapy and Science Center, China Medical University Hospital, China Medical University, Taichung, 40402 Taiwan R.O.C.; 4 Lab of Precision Medicine, Feng-Yuan Hospital, Ministry of Health and Welfare, Taichung, 42055 Taiwan; 5 Department of Colorectal Surgery, China Medical University Hospital, China Medical University, Taichung, 40402 Taiwan; 6 School of Chinese Medicine, China Medical University, Taichung, 40402 Taiwan; 7 Department of Radiation Oncology, China Medical University Hospital, China Medical University, Taichung, Taiwan; 8 Department of Radiation Oncology, School of Medicine, China Medical University, Taichung, 40402 Taiwan; 9 Department of Colorectal Surgery, China Medical University HsinChu Hospital, China Medical University, HsinChu, 302 Taiwan; 10 School of Medicine, China Medical University, Taichung, 40402 Taiwan; 11 Cancer Biology and Precision Therapeutics Center, China Medical University, Taichung, 40402 Taiwan

**Keywords:** Colorectal cancer, Topoisomerase I inhibitor, TLC388, Antitumor immunity, STING

## Abstract

Current immune checkpoint inhibiters (ICIs) have contrasting clinical results in poorly immunogenic cancers such as microsatellite-stable colorectal cancer (MSS-CRC). Therefore, understanding and developing the combinational therapeutics for ICI-unresponsive cancers is critical. Here, we demonstrated that the novel topoisomerase I inhibitor TLC388 can reshape the tumor immune landscape, corroborating their antitumor effects combined with radiotherapy as well as immunotherapy. We found that TLC388 significantly triggered cytosolic single-stranded DNA (ssDNA) accumulation for STING activation, leading to type I interferons (IFN-Is) production for increased cancer immunogenicity to enhance antitumor immunity. TLC388-treated tumors were infiltrated by a vast number of dendritic cells, immune cells, and costimulatory molecules, contributing to the favorable antitumor immune response within the tumor microenvironment. The infiltration of cytotoxic T and NK cells were more profoundly existed within tumors in combination with radiotherapy and ICIs, leading to superior therapeutic efficacy in poorly immunogenic MSS-CRC. Taken together, these results showed that the novel topoisomerase I inhibitor TLC388 increased cancer immunogenicity by ssDNA/STING-mediated IFN-I production, enhancing antitumor immunity for better therapeutic efficacy in combination with radiotherapy and ICIs for poorly immunogenic cancer.

**Supplementary Information:**

The online version contains supplementary material available at 10.1007/s00262-024-03692-8.

## Introduction

Malignant cells undergo evolutionary changes under immunosurveillance, leading to the development of tumors with the ability to escape and suppress the immune system [1]. Currently, effective strategies for overcoming immunosuppression and reinvigorating immune responses involve the use of immune checkpoint inhibitors (ICIs), such as antibodies targeting cytotoxic T lymphocyte antigen 4 (CTLA-4), programmed cell death 1 (PD-1), and programmed cell death 1 ligand 1 (PD-L1) [2]. However, the effectiveness of ICIs is limited to a minority of cancer patients (∼ 20–30%) [2], indicating that solely inhibiting negative signals may not be adequate to enhance therapeutic efficacy in patients lacking sufficient antitumor immunity such as patients with microsatellite-stable colorectal cancer (MSS-CRC).

Numerous preclinical studies have demonstrated that conventional chemotherapeutics, which induce diverse forms of DNA damage and genomic instability, can modify the cancer-immune landscape within the tumor microenvironment by reducing myeloid-derived suppressor cells [3], regulatory T cells (Tregs) [4], as well as the induction of immunogenic cell death (ICD) [5]. Topotecan (TPT), a topoisomerase I (TOP1) inhibitor, has been shown to exhibit immune-enhancing effects through the induction of extensive genomic damage [6–8]. An increase in tumor antigen levels facilitated by TPT significantly upregulates MHC-I expression for antigen presentation [9], enhancing T-cell recognition [10]. Moreover, TPT also triggered the secretion of damage-associated molecular pattern (DAMP) molecules, activating dendritic cells (DCs) through the stimulator of interferon genes (STING)-dependent pathway [11]. These results indicate that the inhibition of topoisomerase I plays a significant role in modulating antitumor immunity, thereby enhancing its therapeutic efficacy [12]. The role of the cyclic GMP-AMP synthase (cGAS)/STING signaling pathway has recently been identified as an important mechanism for antitumor immunity [13]. The cGAS/STING pathway can be activated by cytosolic double-stranded DNA (dsDNA), leading to the activation of TAB-binding kinase 1 (TBK1). TBK1, in turn, activates the transcription factor interferon regulatory Factor 3 (IRF3) to produce type I interferons (IFN-Is). These inflammatory cytokines stimulate DC cross-presentation of tumor antigens, ultimately resulting in the mobilization of tumor-specific CD8^+^ T cells and natural killer (NK) cells, promoting antitumor immunity [14–16]. Therefore, the intrinsic expression of STING is correlated with the activation of IFN-I pathway [17, 18], potentially contributing to DC/T-cell priming and infiltration. Due to their crucial involvement in cancer immune surveillance, STING agonists have emerged as a promising new therapeutic class aimed at enhancing cancer immunogenicity [19, 20]. These agonists have been investigated in preclinical models and have entered clinical trials for the treatment of various solid tumors [21]. However, a major concern in the clinical application of STING agonists is the potential for adverse effects linked to cytokine induction, especially when STING agonists are administered systemically. Systemic administration often requires a higher dose to achieve an effective concentration in tumors, facing challenges related to tumor accessibility and inadequate retention time within tumors.

Lipotecan (TLC388) is a novel liposomal camptothecin that targets topoisomerase I. In our previous studies, we demonstrated that low-dose TLC388 can elicit ICD by releasing HMGB1, annexin A1 (ANXA1), and calreticulin (CRT) to trigger antitumor immunity, augmenting the therapeutic effectiveness of radiotherapy [22]. In this study, we revealed that targeting TOP1 with low-dose TLC388 led to a notable accumulation of cytosolic single-strand DNA (ssDNA). Cytosolic ssDNA accumulation subsequently activates STING/TBK/IRF3 signaling for IFN-I production, which plays a crucial role in the activation of DCs. Additionally, the therapeutic efficacy was significantly enhanced as TLC388 was combined with RT, resulting in complete regression of poorly immunogenic MSS-CRC in vivo. TLC388 plus RT, by enhancing the recruitment of tumor-infiltrating activated dendritic cells, activated CD8^+^ T cell, NK cells, and effector/memory CD8^+^ cells, which sensitized the response to anti-PD1 antibodies. Taken together, these results provide a novel immunotherapeutic potential by targeting TOP1 with TLC388 maximizes the clinical benefit to poorly immunogenic CRC patients in combination with radiotherapy as well as immunotherapy.

## Materials and methods

### Cell culture

Colorectal cancer cell lines, including SW480 (CCL-228 ™), SW620 (CCL-227 ™), HCT116 (CCL-247™), HT29 (HTB-38), CoLo320DM (CCL-220), and CT26 (CRL-2638), were obtained from the American Type Culture Collection (ATCC). These cells were cultured and maintained in RPMI 1640 medium supplemented with 10% fetal bovine serum (Life Technologies, Grand Island, New York, USA), 2 mM glutamine, 100 U/ml penicillin, 100 mg/ml streptomycin, and 1 mM pyruvate at 37 °C in a humidified atmosphere with 5% CO_2_.

For the experiment, SW480, SW620, HCT116, HT29, CoLo320DM, and CT26 cells were seeded in 6-cm dishes at approximately 80% confluence in RPMI 1640 supplemented with 10% FBS on the day before treatment. The cells were then harvested for western blot analysis at the indicated time points.

### Antibodies and Western blot analysis

The antibodies used in this study included the following: anti-cleaved caspase-3 (#9661, Cell Signaling Technology and IR96-401, iReal Biotech. ), anti-GAPDH (IR3-8, iReal Biotech. ), anti-cGAS (ab224144, Abcam), anti-p-STING (AP1199, ABclonal), anti-STING (A20175, ABclonal), anti-p-TBK1 (AP1026, ABclonal), anti-TBK1 (A2573, ABclonal), anti-p-IRF3 (AP0623, ABclonal), anti-IRF3 (#41,074, Cell Signaling Technology), and anti-PD-L1 (ab205921, Abcam and AP17952-1, Proteintech) were used. All secondary antibodies (HRP-conjugated anti-rabbit, anti-mouse and anti-goat) were obtained from Santa Cruz Biotechnology.

Total lysates (30 µg) were separated on an SDS–PAGE gel and subsequently transferred onto PVDF membranes (Millipore, MA, USA) [23, 24] for immunoblot analyses using the indicated antibodies overnight at 4 °C. The membranes were subsequently probed with HRP-conjugated secondary antibodies for 2 h at room temperature. All the antibodies were diluted in T-Pro Protein Free Blocking Buffer (BioLion Tech., Taipei, Taiwan). The membrane was then treated with Immobilon Western Chemiluminescent HRP Substrate (Millipore, CA, USA), visualized using an ImageQuant™ LAS 4000 biomolecular imager (GE Healthcare, Amersham, UK), processed using Adobe Photoshop and quantified using ImageJ software (NIH, MD, USA). To ensure accurate analysis, each blot was stripped with immunoblotting stripping buffer (BioLion Tech.) before subsequent incubation with other antibodies.

### qRT–PCR

Total RNA was extracted from cell lines with TRIzol (Invitrogen, CA, USA), quantitated by measuring the absorbance at 260 nm, and then reverse transcribed into cDNA using iScript™ Reverse Transcription Supermix (Bio-Rad, CA, USA) following the manufacturer’s instructions [25, 26]. Primers were designed using the Primer Design Tool (NCBI, USA) based on sequence information from the NCBI database. qRT–PCR was carried out in a final reaction volume of 20 µL with iQ™ SYBR® Green Supermix (Bio-Rad, CA, USA) using the CFX96 Touch Real-Time PCR Detection System (Bio-Rad). Each sample was analyzed in triplicate, and GAPDH was used as a reference gene for normalization. Relative gene expression levels were calculated using the 2^−ΔΔCt^ method, and comparisons between gene expression levels were made using the *t test*.

### Evaluation of the immune cell profiles induced by TLC388 and local RT in vivo

BALB/c mice (female, 4 weeks old) were housed following the institutional guidelines approved by the China Medical University Institutional Animal Care and Use Committee. In brief, CT26 cells (3 × 10^5^ cells/mouse) were suspended in 100 µL of 50% Matrigel and subcutaneously inoculated into the right legs of each mouse. After 7 days, TLC388 (2.5 mg/kg/mouse, intraperitoneal injection) was administered and local radiotherapy was given (5Gy). Tumor volume was measured every 3 days throughout the study. The longest and shortest diameters (L and W, respectively) of the tumors were measured using Vernier calipers every 3 days, and tumor volume (V) was calculated by the Formula V = (L ×W^2^)/2. At the conclusion of the experiment, the mice were sacrificed, and tumor tissues were collected and subjected to immunofluorescent staining.

### Immunofluorescent staining

The antibodies used in this study were as follows: anti-CD11c (ab52632, Abcam) and anti-mouse granzyme B (ab255598, Abcam). The tissue slides were deparaffinized and subjected to heat-mediated antigen retrieval with Antigen Unmasking Solutions (H3300, Vector Laboratories, Burlingame, CA). Incubate tissue slides with 5% horse serum for 10 min. Tissue Sect. (3 μm thick) were stained with indicated primary antibodies and the PE-conjugated secondary antibodies, and counterstained with DAPI [27, 28].

Staining for immune cells was positive when detected in the tumor-infiltrating lymphocytes (TILs) and was evaluated using a microscope (OLYMPUS BX53, Tokyo, Japan). For the detection of TILs, the tissue was viewed at 40× magnification, and the area with the highest density of CD11C^+^ and GzmB^+^ TILs within the malignant cells was counted at 400× magnification (no. of TILs/high-power field). The average number of tumor-infiltrating immune cells in five high-power fields was included in the evaluation [29–31].

### Co-culture assay

The human monocytic leukemia cell line THP-1 was cultured and maintained in RPMI 1640 medium supplemented with 10% FBS, 2 mM glutamine, 1 mM sodium pyruvate, 1% P/S. THP-1 cells were differentiated into immature DCs with 1500 IU/ml rhIL-4 (Sino Biological, Beijing, China) and 1500 IU/ml rhGM-CSF (Sino Biological, Beijing, China) treatment in culture medium for at least 7 days [32]. The cytokine-supplemented culture medium was refreshed every 2–3 days. After treatment with TLC388 (0.5µM) for 24 h, HT29 cells were co-cultured with THP-1 iDCs for 24 h. The co-cultured THP-1 iDCs with TLC388-treated HT29 cells were carried out separately in 0.4-µM pore sized filter transwell insert on a 6-well culture plate. The phenotype of the THP-1 iDCs were then analyzed by flow cytometry analysis.

### Combinational therapies of TLC388, local radiotherapy and anti-PD-1 blockade in vivo

A total of 3 × 10^5^ CT26 cells in 100 µl of 50% Matrigel were inoculated into the right flanks of BALB/c mice. The treatments were initiated on Day 7 after tumor inoculation: TLC388 (intraperitoneal injection, 2.5 mg/kg/mouse for 3 consecutive days). On Days 7 and 14, the mice received radiotherapy (5 Gy), and an anti-mouse PD-1 antibodies was administered on Days 11, 14 and 17 (5 mg/kg, intraperitoneal injection, 4 times with 3-day intervals between injections; Bio×Cell clone RMP1-14, NH, USA). The longest and shortest diameters (L and W, respectively) of the tumors were measured using Vernier calipers (Sata, Shanghai, China) every 3 days, and tumor volume (V) was calculated using the following formula: V = (L ×W^2^)/2. At the conclusion of the experiment, the mice were sacrificed, and tumor tissues were collected for lysis and flow cytometry, and subjected to immunofluorescent staining and qRT-PCR.

### Flow cytometry analysis of immune cell profiles

Tumors were dissected from the mice, weighed and then placed in petri dishes containing blank RPMI media at room temperature to prevent dehydration. The tumors were minced into small pieces (1–2 mm) with a beaver blade, filtered through a 70 μm strainer, centrifuged, and then resuspended in blank RPMI media. Thereafter, the cell suspensions were layered over Ficoll-Paque media and centrifuged at 1,025 × g for 20 min. The layer of mononuclear cells was transferred to a conical tube, 20 ml of complete RPMI media was added, the mixture was gently mixed, and the sample was centrifuged at 650 × g for 10 min twice. Finally, the supernatant was removed, and the TILs were resuspended in complete RPMI media.

Then, the TILs were resuspended in 500 µL of staining buffer (2% BSA and 0.1% NaN_3_ in PBS). The cells were stained with different surface marker panels: (1) CD8^+^ T cells: CD45-PE (E-AB-F1136UD, Elabscience, Texas, USA) and CD8a-PerCP (E-AB-F1104UF, Elabscience, Texas, USA); (2) Foxp3^+^ regulatory T cells: CD45-PE (E-AB-F1136UD, Elabscience, Texas, USA), CD4-APC (E-AB-F1097UE, Elabscience, Texas, USA), CD25-PerCP (E-AB-F1194J, Elabscience, Texas, USA) and Foxp3-FITC (E-AB-F1238C, Elabscience, Texas, USA). For intracellular staining, TILs were fixed and permeabilized with a Foxp3/transcription factor staining buffer set (eBioscience, Thermo Fisher, CA, USA) after cell surface staining. The cells were then stained with Foxp3-FITC for 45 min. The samples were washed twice with Perm Wash Buffer and analyzed with a BD Canto II flow cytometer (BD, CA, USA). Isotype controls, namely, the PerCP-conjugated rat IgG2b κ isotype control (E-AB-F09842J), APC-conjugated rat IgG2b (E-AB-F09843E, Elabscience, Texas, USA), and PE-conjugated rat IgG2b κ isotype control (E-AB-F09842D, Elabscience, Texas, USA), were used [18].

### Tissue microarray (TMA) construction for immunohistochemistry

Colorectal cancer patients who were diagnosed and treated between 2011 and 2014 at China Medical University Hospital were enrolled in our cohort [29, 33, 34]. The TMA included resected primary tumor tissue and corresponding normal mucosa specimens, which was approved by the Institutional Review Board (IRB) of China Medical University Hospital [Protocol number: CMUH107-REC2-008].

IHC was performed using 3-µm-thick TMA sections with the indicated antibodies (anti-human STING #13,647; Cell Signaling Technology), followed by incubation with the HRP-conjugated avidin biotin complex (ABC) Kit (Vector Laboratories, CA, USA) and the HRP substrate DAB chromogen (Vector Laboratories) and counterstaining with hematoxylin [30, 35].

The tumor STING expression was evaluated and scored based on the intensity and percentage of cells positive for the histoscore (H-score), which was calculated by performing a semiquantitative assessment of both the intensity of the staining (0: negative staining; 1: weak; 2: moderate; and 3: strong staining) and the percentage of immunopositive cells. The H-score ranged from 0 to 300 and we categorized it into low or high group base on the average H-score [23, 31].

### Statistical analysis

All the experiments were conducted at least 3 times. All the statistical analyses were performed using GraphPad Prism 7 statistical software (GraphPad Software, CA, USA) [35]. The data were analyzed using two-way ANOVA followed by Bonferroni post hoc correction, one-way ANOVA followed by Dunnett’s post hoc test, or an unpaired t test where appropriate. The data are presented as the mean ± SEM. Student’s t test was used to compare the differences in tumor sizes and positive cell counts between the two groups. ANOVA was used for comparisons of the results involving combinations of TLC388, RT, and PD-1 blockade among the groups. *p* < 0.05 was considered to indicate a significant difference. The survival period was defined as the time from surgery to cancer-specific death, and cancer-specific survival (CSS) was assessed by Kaplan‒Meier survival analysis.

## Results

### The novel liposomal camptothecin TLC388 enhanced STING activation compared with that of other topoisomerase I inhibitors in CRC

**Fig. 1:**
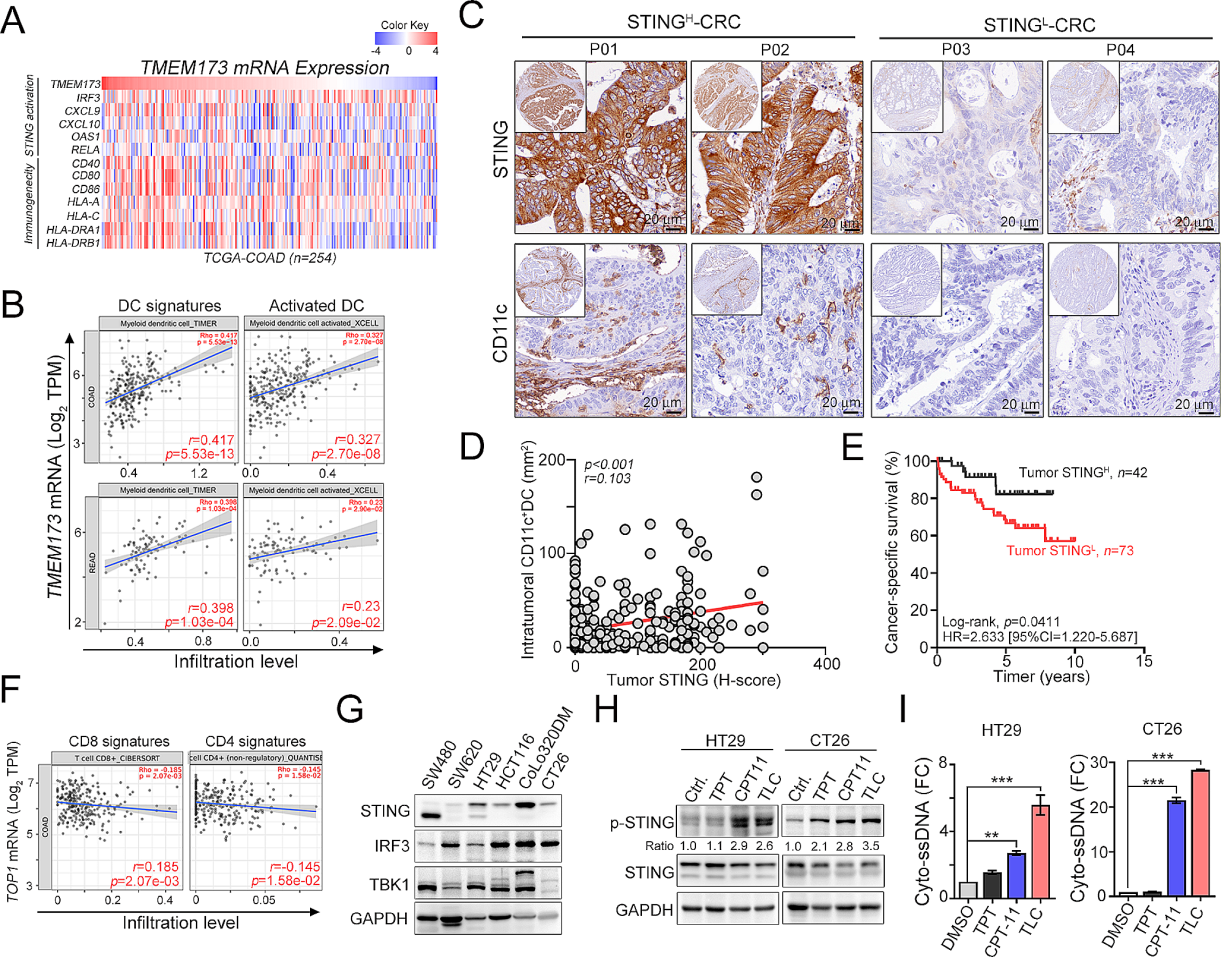
TLC388 significantly increased the STING-dependent pathway by promoting ssDNA accumulation in CRC. (**A**) The association between *TMEM173* (*STING)* signature and cancer immunogenicity-related gene signature were analyzed. The gene expression data were retrieved from TCGA-COAD (*n* = 254). (**B**) The association between *TMEM173* (*STING*) and dendritic cells signatures were analyzed in TCGA-COAD cohort (*n* = 458) on TIMER2.0 website (http://timer.comp-genomics.org/timer/). (**C**) The representative images of tumor STING and tumor-infiltrating CD11c^+^ DCs in colorectal cancer patients (*n* = 259). (**D**) The relationship between tumor STING and infiltration of dendritic cells was examined in colorectal cancer patients (*r* = 0.103, *n* = 259, *p* < 0.001). (**E**) High tumor STING expression was associated with favorable CSS in colorectal cancer patients who received post-operative chemotherapy (*n* = 115, Log-rank *p* = 0.0411). (**F**) The representative images of tumor STING and tumor-infiltrating CD8 and CD4 signatures in TCGA-COAD cohort (*n* = 458) on TIMER2.0 website (http://timer.comp-genomics.org/timer/). (**G**) The total protein expression of STING in colorectal cancer cell lines. (**H**) HT29 and CT26 cells were treated with different topoisomerase I inhibitors topotecan (TPT, 0.5 µM), irinotecan (CPT11, 0.5 µM), and TLC388 (0.5 µM) for 24 h and analyzed by western blotting. (**I**) HT29 and CT26 cells were treated with TLC388 (0.5, 1 and 2.5 µM) for 24 h. The cytosolic fraction was isolated for single-stranded DNA analysis (*n* = 3). These data were obtained from three independent experiments, and the values represent the means ± S.D. One-Way ANOVA *t*-test

The cancer-intrinsic cGAS/STING signaling pathway plays a critical role in tumor suppression and immune surveillance through the activity of the IFN-I pathway [17, 18, 24, 36]. Frequent defects in cGAS/STING-dependent signaling pathways in CRC contribute to impaired endogenous T-cell priming and infiltration, affecting antitumor immunity [17, 18, 36, 37]. Indeed, we observed a positive correlation between the level of *TMEM173/STING* mRNA and the IFN-I signature, as well as cancer immunogenicity-related genes (Fig. 1A), based on data retrieved from The Cancer Genome Atlas colorectal adenocarcinoma (TCGA-COAD) cohort. Additionally, the level of *TMEM173/STING* mRNA was positively correlated with the infiltration levels of general and activated dendritic cell signatures (Fig. 1B). These results implied that the activation of cancer-intrinsic STING may enhance cancer immunogenicity, leading to increased infiltration of dendritic cells (DCs) for T-cell priming and activation. By evaluating cancer-intrinsic STING with IHC in a retrospective cohort (*n* = 259), we found lower expression levels of STING in patients with CRC (*n* = 259; Fig. 1C). Notably, 58.3% of the CRC patients did not exhibit STING expression in cancer cells (151/259 = 58.3%, Fig. 1C). Additionally, the level of cancer-intrinsic STING was positively correlated with the infiltration of CD11c^+^ DCs in CRC patients (Fig. 1C and D, *p* < 0.001, *r* = 0.103, *n* = 259). Low STING expression on cancer cells correlated with shorter cancer-specific survival (CSS) in patients with CRC who underwent postoperative adjuvant chemotherapy (Fig. 1E and Table S1, log-rank *p* = 0.0411, *n* = 115).

Among the commonly used chemotherapeutic agents in CRC patients, topoisomerase 1 (TOP1) inhibitors have been shown to promote the maturation of dendritic cells, thereby contributing to antitumor immunity through a STING-dependent pathway [11, 38, 39]. This effect is attributed to the extensive genomic damage caused by TOP1 inhibitors [8, 11, 12]. We found that the expression level of *TOP1* mRNA was negatively associated with the infiltration level of the CD8 signature (*r*=-0.185, *p* = 0.00207) and the CD4 signature (*r*=-0.145, *p* = 0.0158, Fig. 1F). However, there was no significant correlation between the *TOP2A*/*TOP2B* mRNAs and immune cell signatures (Fig. S1A). These results suggest that targeting TOP1 but not TOP2 might remodel the tumor microenvironment and enhance therapeutic efficacy in CRC. Therefore, we examined the impact of various TOP1 inhibitors on STING activation in CRC cells (Fig. 1G and H). We observed that TOP1 inhibitors induced different extents of STING activation, including topotecan (TPT), camptothecin (CPT11) and liposomal camptothecin (lipotecan, TLC388, Fig. 1H). Notably, compared with other TOP1 inhibitors, TLC388 exhibited a greater capacity to enhance STING phosphorylation in MSS-CRC cell lines (HT29 and CT26), which are characterized by low immunogenicity (Fig. 1H). Furthermore, we found that the cytosolic accumulation of ssDNA resulting from TLC388 treatment was significantly increased (Fig. 1I). These results suggest that TLC388 effectively induces the accumulation of cytosolic ssDNA, which arises from genomic damage and promotes cGAS/STING activation in CRC cells.

### Low-dose TLC388 markedly increased STING activation in poorly immunogenic MSS-CRC cells, eliciting DC maturation to facilitate a T-cell-mediated immune response

**Fig. 2:**
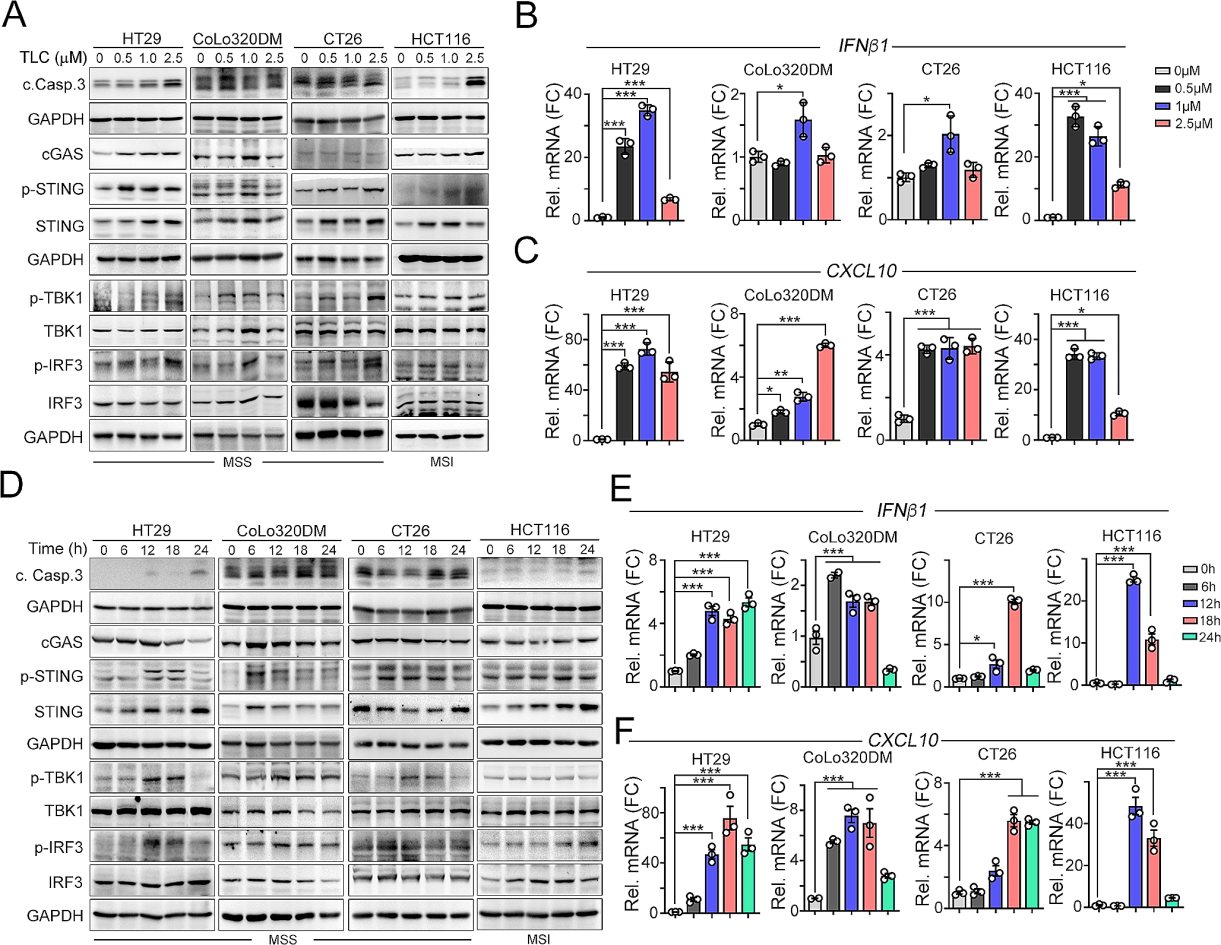
Low-dose TLC388 enhances STING-mediated IFN-I production. (**A**) HT29, CoLo320DM, CT26 and HCT116 cells were treated with diverse concentrations of TLC388 for 24 h. The level of different proteins was analyzed by western blotting. (**B**) HT29, CoLo320DM, CT26 and HCT116 cells were treated with diverse concentrations of TLC388 for 24 h. The level of *IFNβ1* mRNA was analyzed by qRT-PCR (*n* = 3). *p < 0.05 and **p < 0.01. One-Way ANOVA *t*-test. (**C**) HT29, CoLo320DM, CT26 and HCT116 cells were treated with diverse concentrations of TLC388 for 24 h. The mRNA level of *CXCL10* was analyzed by qRT-PCR (*n* = 3). *p < 0.05, **p < 0.01 and ***p < 0.001. These data were obtained from three independent experiments, and the values represent the means ± S.D. One-Way ANOVA *t*-test. (**D**) HT29, CoLo320DM, CT26 and HCT116 cells were treated with different time point of TLC388 (0.5 µM). The level of different proteins was analyzed by western blotting. (**E**) HT29, CoLo320DM, CT26 and HCT116 cells were treated with different time point of TLC388 (0.5 µM). The level of *IFNβ1* mRNA was analyzed by qRT-PCR (*n* = 3). *p < 0.05 and **p < 0.01. One-Way ANOVA *t*-test. (**F**) HT29, CoLo320DM, CT26 and HCT116 cells were treated TLC388 (0.5 µM) at various time point. The mRNA level of *CXCL10* was analyzed by qRT-PCR (*n* = 3). *p < 0.05 and **p < 0.01. One-Way ANOVA *t*-test

The majority of CRC patients (∼ 85%) exhibit unresponsiveness to ICIs due to the low immunogenicity of MSS-CRC [40]. Therefore, we assessed whether TLC388 could enhance STING activation in both MSS-CRC cell lines (HT29, CoLo320DM, and CT26) and MSI-CRC cell line (HCT116). We observed the level of the apoptotic cell marker caspase-3 and STING/TBK1/IRF3 activation was increased with dose-dependent manner in both MSS-CRC cell lines (HT29, CoLo320DM, and CT26) and MSI-CRC cell line (HCT116, Fig. 2A). The proinflammatory cytokines *IFNβ1* and *CXCL10* were also elicited by low-dose TLC388 (0.5-1.0 µM, Fig. 2B and C), indicating that TLC388 has a superior ability to activate STING-mediated IFN-I production, thereby enhancing cancer immunogenicity, especially in cells with low immunogenicity. Moreover, treatment with low-dose TLC388 remarkably induced STING phosphorylation in time-dependent manner (Fig. 2D). Notably, the phosphorylation of STING and its downstream proteins peaked at 18 h after TLC388 treatment in all cell lines (Fig. 2D). The production of the proinflammatory cytokines *IFNβ1*, *CXCL10*, and *IL12A* also peaked at 18 h after TLC388 treatment (Fig. 2E and F, and Fig. S1B). These results indicated that low-dose TLC388 significantly promoted cGAS/STING activation in CRC, including in patients with low immunogenic MSS-CRC.

**Fig. 3:**
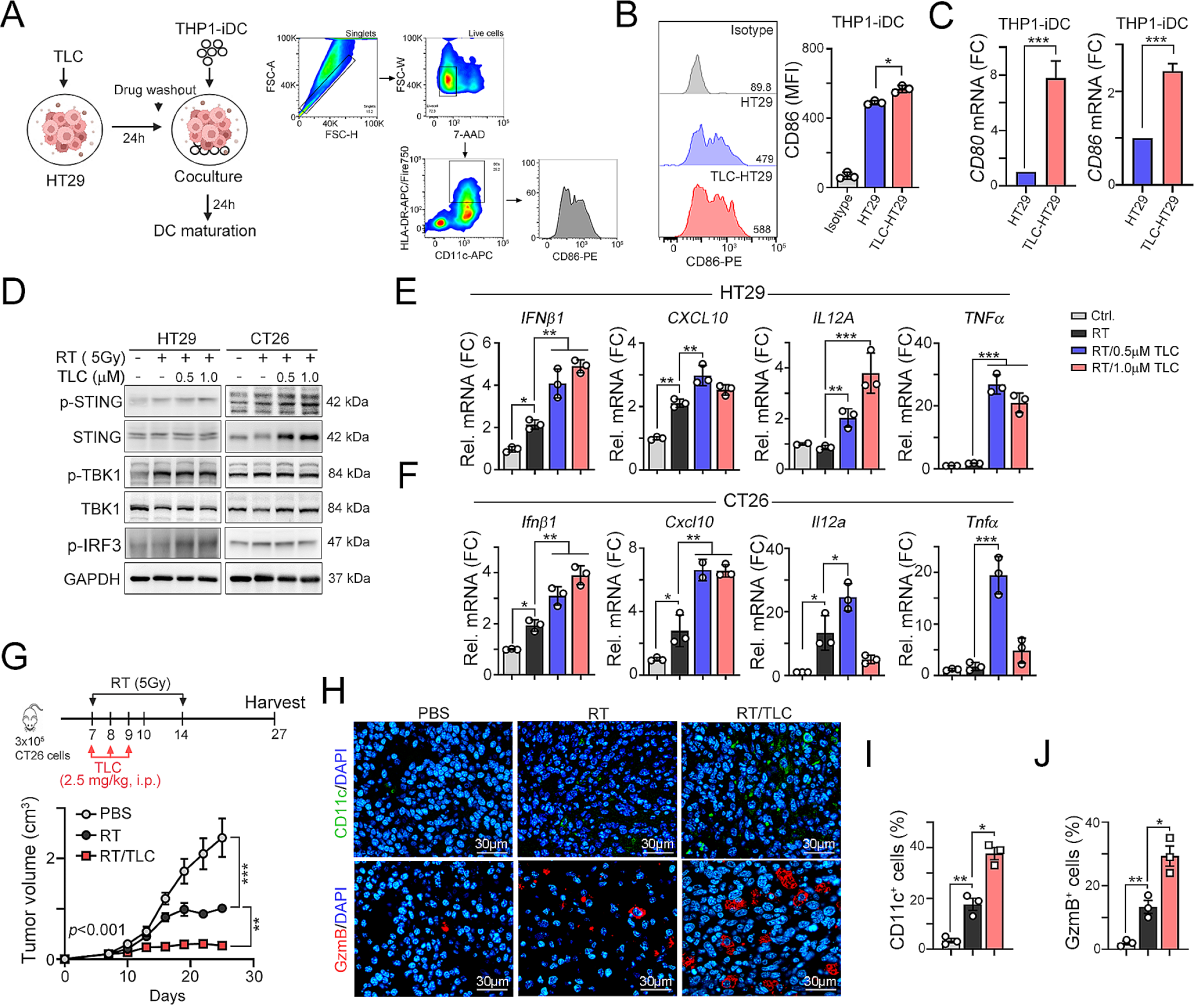
TLC388 synergistically enhanced RT-induced cancer immunogenicity. (**A**) The schematic diagram of immature DC cocultured with TLC388-treatment HT29 cell and analyzed DC maturation marker by flow cytometry. (**B**) The TLC388 (0.5 µM)-treated HT29 cells were cocultured with THP1-iDC cells, which were differentiated into immature DC (iDC) by IL-4 (1500 IU/ml) and GM-CSF (1500 IU/ml) for 7 days, for 24 h. The DC marker (CD86) was evaluated by flow cytometry. **p < 0.01. One-way ANOVA *t*-test. (**C**) HT29 cells were treated with TLC388 (0.5 µM) for 24 h, and then washout the drugs to coculture with THP1-iDC for 24 h. The mRNA level of DC markers (*CD86* and *CD80*) was determined by qRT-PCR(*n* = 3). *p < 0.05 and **p < 0.01. One-Way ANOVA *t*-test. (**D**) HT29 and CT26 cells were irradiated for 5 Gy and treated with TLC388 (0.5 and 1 µM) for 24 h. The cell lysate was harvested for western blot analysis. (**E**) HT29 cells were irradiated with 5 Gy and treated with TLC388 (0.5 and 1 µM) for 24 h. The level of *IFNβ1, CXCL10*, *IL12a*, and *TNFα* mRNA was analyzed by qRT-PCR (*n* = 3). *p < 0.05 and **p < 0.01. One-way ANOVA *t*-test. (**F**) CT26 cells were irradiated with 5 Gy and treated with TLC388 (0.5 and 1 µM) for 24 h. The level of *IFNβ1, CXCL10*, *IL12a*, and *TNFα* mRNA was analyzed by qRT-PCR (*n* = 3). *p < 0.05 and **p < 0.01. One-way ANOVA *t*-test. (**G**) Tumor growth of CT26-driven colon carcinoma established in BALC/c mice (*n* = 6 per group) that were treated with RT (5 Gy for 2 fractions) and RT/TLC (TLC:2.5 mg/kg). Tumor growth is reported as the mean tumor volume ± SD. *p < 0.05 and **p < 0.01. CR: complete response. Two-way ANOVA *t*-test. (**H**) The tumor-infiltrating CD11c^+^ DC and GzmB^+^ immune cells within resected tumors were analyzed by immunofluorescent staining (*n* = 3). (**I**) The quantification of tumor-infiltrating CD11c^+^ dendritic cells within resected tumors (*n* = 3). *p < 0.05 and **p < 0.01. One-way ANOVA *t*-test. (**J**) The quantification of tumor-infiltrating GzmB^+^ T cells within resected tumors (*n* = 3). *p < 0.05 and **p < 0.01. One-way ANOVA *t*-test

We further assessed whether the activation of STING in CRC cells could enhance dendritic cell activation. HT29 cells were treated with low-dose TLC388 (0.5 µM) and PBS (vehicle) for 24 h and subsequently cocultured with THP1-iDCs for 24 h (Fig. 3A). In line with the STING-mediated expression of proinflammatory cytokines, the co-culture of THP1-iDCs with HT29 cells treated with TLC388 led to the upregulation of the costimulatory molecules CD86 and CD80 (Fig. 3B and C), which are indicative of DC maturation. These results collectively suggest that STING-mediated induction in MSS-CRC cells by TLC388 promotes the maturation of DCs, highlighting the potential of TLC388 to augment the priming of tumor antigen-specific T-cell responses.

### TLC388 reshaped the tumor microenvironment to eradicate cancer cells in combination with local radiotherapy in vivo

Irradiation also induces various forms of DNA damage and genomic instability [15], activating the cGAS/STING pathway and triggering antitumor immune responses [18]. To determine whether TLC388-induced enhancement of cancer immunogenicity was synergistically increased in combination with RT, we treated cells with low-dose TLC388 and subjected them to irradiation. As shown in Fig. 3D, we observed a significant increase in STING and TBK1 phosphorylation by RT plus TLC388 in MSS-CRC cells. Additionally, the level of proinflammatory cytokines *IFNβ1*, *CXCL10*, *IL12A*, and *TNFα* was profoundly increased after RT/TLC388 treatment, compared to RT alone (Fig. 3E and F). These results indicated that combination treatment with low-dose TLC388 and radiotherapy significantly upregulated cancer immunogenicity in poorly immunogenic CRC cells.

Our previous studies demonstrated that moderate TLC388 (5 mg/kg) also induced immunogenic cell death (ICD), increasing the therapeutic efficacy of radiotherapy through the release of HMGB1 and ANXA1 in vitro and in vivo [22]. Therefore, we optimized the dose of TLC388 in combination with local radiotherapy to examine its therapeutic efficacy in CT26-bearing BALB/c mice (MSS-CRC, Fig. 3G). We found that low-dose TLC388 (2.5 mg/kg) significantly enhanced the therapeutic efficacy of RT, resulting in delayed tumor growth (Fig. 3G). Moreover, the density of tumor-infiltrating CD11c^+^ DCs and GzmB^+^ T cells was significantly increased in the RT plus TLC388 group (Fig. 3H and I, and 3J). Taken together, these results demonstrated that TLC388 reshaped the tumor microenvironment to eradicate poorly immunogenic cancer cells when combined with local radiotherapy.

### TLC388 upregulated cancer-intrinsic PD-L1 expression and increased susceptibility to ICIs in combination with local radiotherapy in poorly immunogenic MSS-CRC

**Fig. 4:**
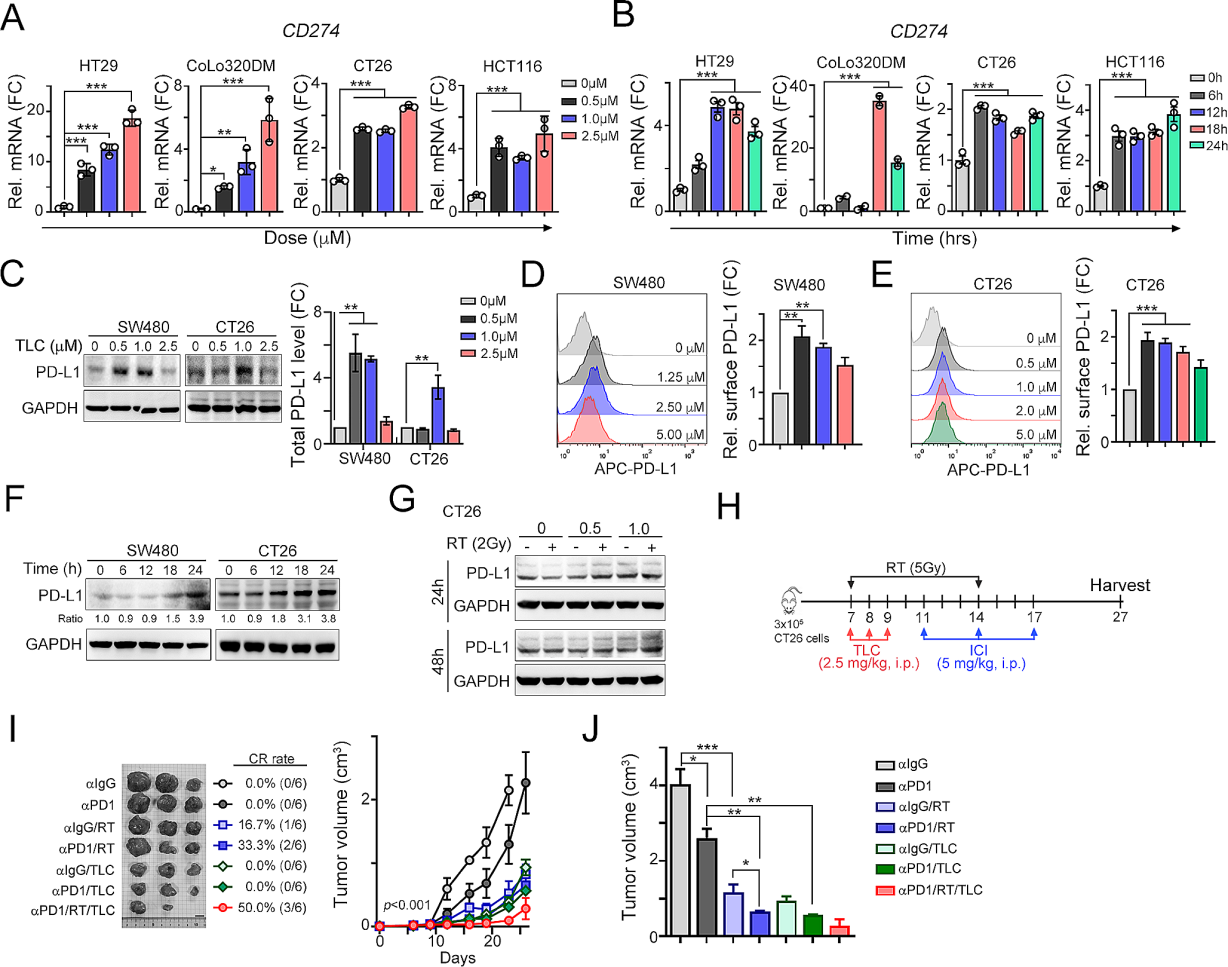
TLC388 promotes PD-L1 upregulation and increases the therapeutic efficacy of ICIs in combination with radiotherapy in vivo. (**A**) HT29, CoLo320DM, CT26 and HCT116 cells were treated with diverse concentrations of TLC388 for 24 h. The level of *CD274* mRNA was analyzed by qRT-PCR (*n* = 3). *p < 0.05, **p < 0.01, and ***p < 0.001. One-way ANOVA *t*-test. (**B**) HT29, CoLo320DM, CT26 and HCT116 cells were treated with different period of TLC388 (0.5 µM). The mRNA level of *CD274* was analyzed by qRT-PCR (*n* = 3). ***p < 0.001. One-way ANOVA *t*-test. (**C**) SW480 and CT26 cells were treated with diverse concentrations of TLC388 for 18 h. The total protein expression level of PD-L1 was analyzed by western blotting (*n* = 3). **p < 0.01. One-way ANOVA *t*-test. (**D**) SW480 cells were treated with various concentrations of TLC388 for 18 h, the surface level of PD-L1 was subsequently analyzed by flow cytometry (*n* = 3). **p < 0.01. One-way ANOVA *t*-test. (**E**) CT26 cells were treated with diverse concentrations of TLC388 for 18 h. The surface level of PD-L1 was subsequently analyzed by flow cytometry (*n* = 3). ***p < 0.001. One-way ANOVA *t*-test. (**F**) SW480 and CT26 cells were treated at various time point of TLC388 (0.5 µM). The total protein level of PD-L1 was analyzed by western blotting (*n* = 3). (**G**) CT26 cells were irradiated with 2 Gy and treated with TLC388 (0.5 and 1 µM) for 24 and 48 h. The protein expression level of PD-L1 was analyzed by western blotting. (**H**) The treatment regimen involving TLC388, RT, and ICIs was implemented as follows: BALC/c mice were subcutaneously injected with CT26 cells for 7 days (*n* = 6 per group). Subsequently, the mice were randomly allocated into subgroups for specific treatments. Local RT (5 Gy for 2 fractions), TLC388 (2.5 mg/kg, i.p.), and anti-mouse PD-1 antibodies (5 mg/kg, i.p.) were administered on designated days. (**I**) Tumor volume was measured every three day and presented as the mean tumor volume ± SD. *p < 0.05 and **p < 0.01. Two-way ANOVA *t*-test. CR: complete response. (**J**) The tumor volume was evaluated on Day 27 (*n* = 3). *p < 0.05, **p < 0.01, and ***p < 0.001. One-way ANOVA *t*-test

Genotoxic chemotherapies, including TOP1 inhibitors, have been shown to upregulate tumor PD-L1 expression and confer clinical benefits when combined with ICIs [41, 42]. Therefore, we investigated whether TLC388 can directly increase tumor PD-L1 expression. As shown in Fig. 4A and B, TLC388 treatment exhibited significantly increased *CD274* (PD-L1) mRNA expression in these cell lines with dose-dependent and time-dependent manner. Furthermore, the total and surface PD-L1 levels were also upregulated in dose-dependent and time-dependent manner (Fig. 4C, D and E, and 4F). Combined treatment with RT and TLC388 led to greater PD-L1 upregulation (Fig. 4G), suggesting that TLC388 may increase susceptibility to ICIs in an ICI-unresponsive MSS-CRC model. Therefore, we treated CT26-bearing BALB/c mice (MSS-CRC) with low-dose TLC388, local radiotherapy, and anti-mouse PD-1 antibodies (Fig. 4H). As shown in Fig. 4I, we found that the response to ICIs alone was unsatisfactory, with a decrease in tumor volume of approximately 30%. In combination with TLC388, there was a significant decreased in tumor volume (2.27±0.49 cm^3^ vs. 0.56±0.02 cm^3^, *p* < 0.001; Fig. 4I and J). Moreover, local radiotherapy also increased the response to ICIs (2.27±0.49 cm^3^ vs. 0.64±0.03 cm^3^, *p* < 0.001; Fig. 4I). Compared with RT alone, RT plus ICIs also led to a 33.3% complete response (2/6) (16.7%, 1/6; Fig. 4I). Furthermore, triple treatment resulted in greater extents of tumor regression and complete response (50%, 3/6; Fig. 4I and J).

**Fig. 5:**
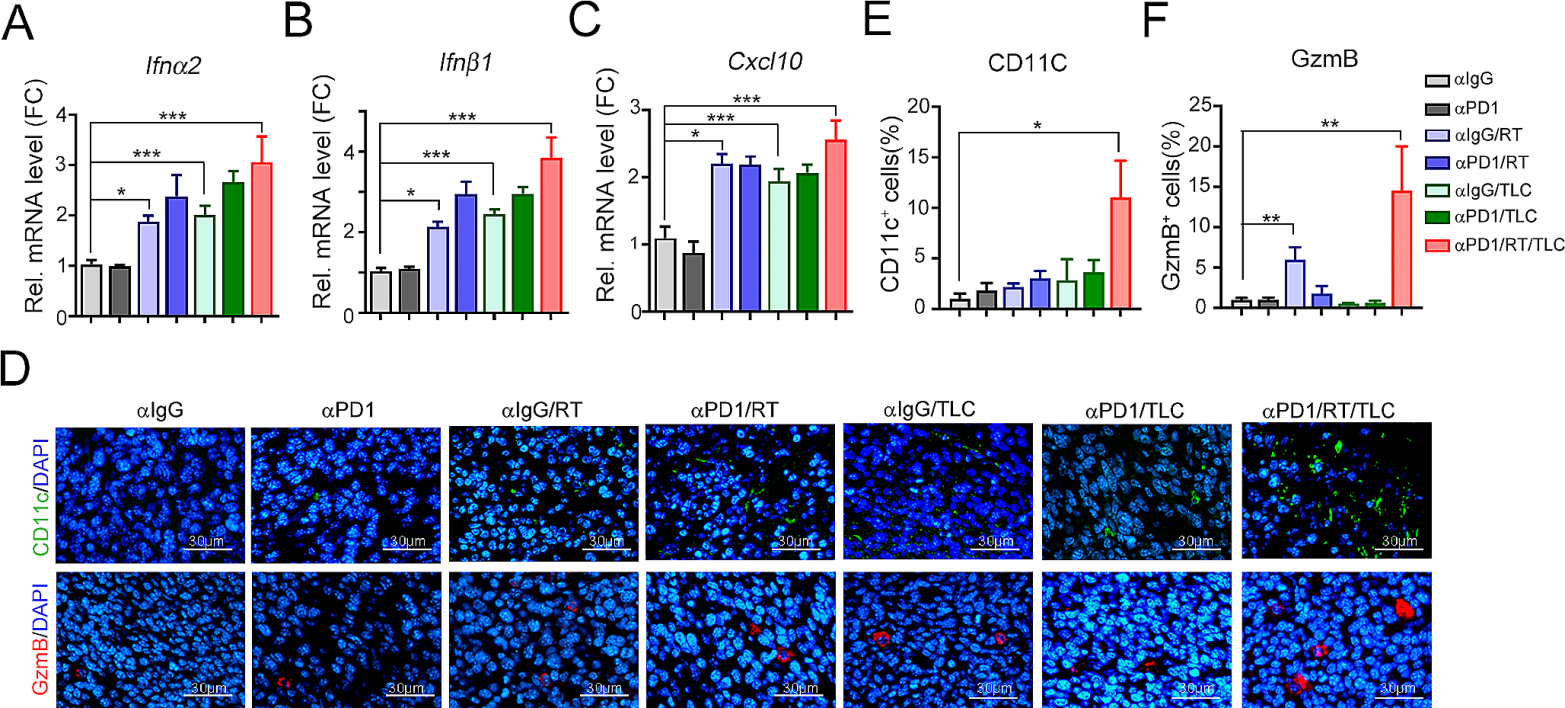
Combinational therapies markedly enhanced type I IFN production and immune cell infiltration in vivo. (**A**) The resected tumors were homogenized, and the expression level of *Ifnα2* was analyzed by qRT-PCR (*n* = 3). *p < 0.05 and ***p < 0.001. One-way ANOVA *t*-test. (**B**) The resected tumors were homogenized, and the expression level of *Ifnβ1* was analyzed by qRT-PCR (*n* = 3). *p < 0.05 and ***p < 0.001. One-way ANOVA *t*-test. (**C**) The resected tumors were homogenized, and the expression level of *Cxcl10* resected tumors was analyzed by qRT-PCR (*n* = 3). *p < 0.05 and ***p < 0.001. One-way ANOVA *t*-test. (**D**) The representative results were infiltration of CD11C^+^ DCs and GzmB^+^ immune cells were analyzed by immunofluorescent (*n* = 3). (**E**) The density of CD11C^+^ DCs was quantified under high-power-field microscopy (*n* = 3). *p < 0.05. One-way ANOVA *t*-test. (**F**) The density of GzmB^+^ T cells was quantified under high-power-field microscopy (*n* = 3). The quantitative analysis of the immunofluorescent results. **p < 0.01. One-way ANOVA *t*-test

**Fig. 6:**
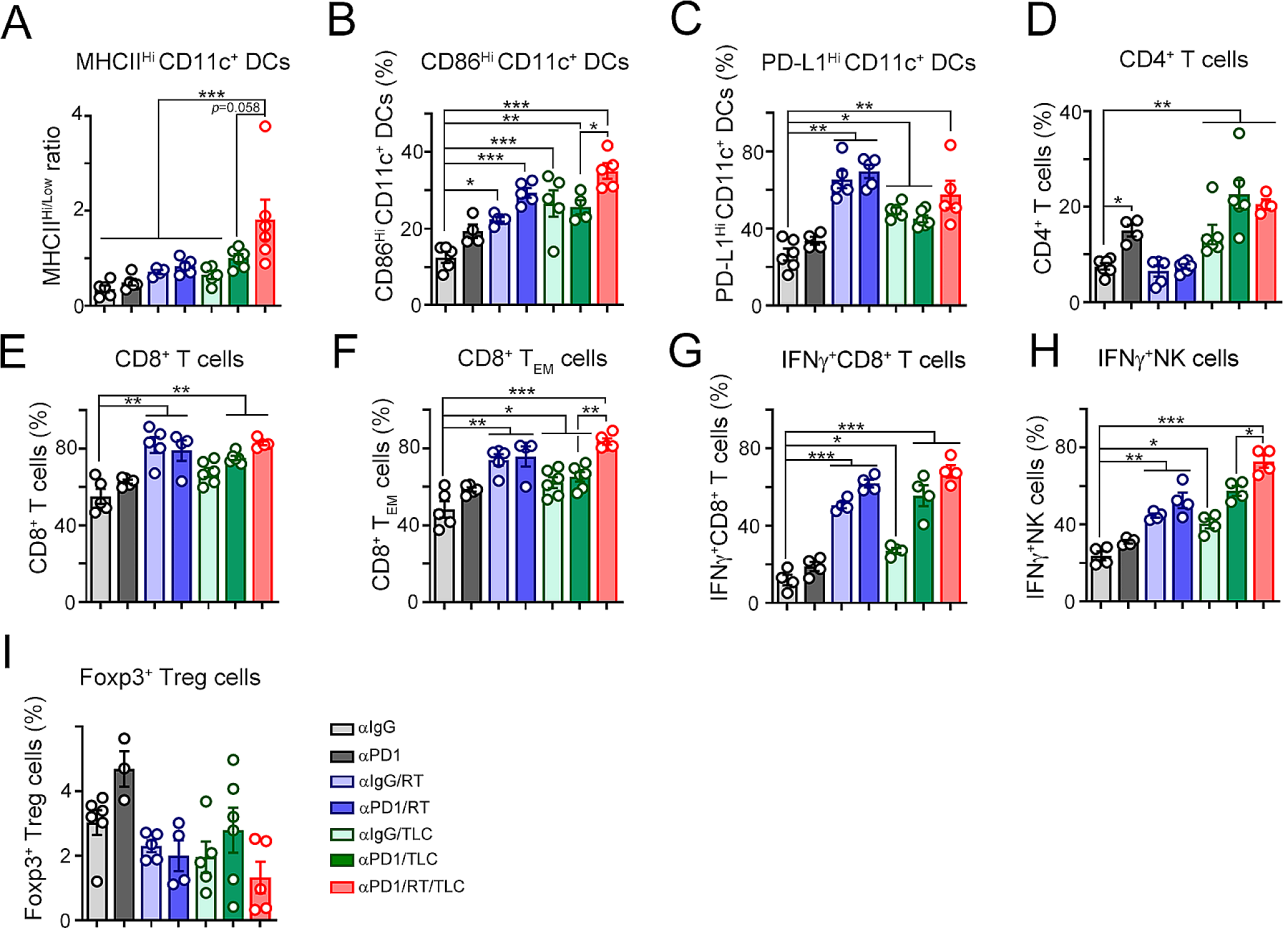
Combination therapies significantly alter the tumor microenvironment, leading to the recruitment of dendritic cells, functional CD8^+^ T cells and natural killer (NK) cells. (**A**) The ratio of tumor-infiltrating MHC-II^Hi^ CD11c^+^ DCs in resected tumors was analyzed by flow cytometry (*n* = 3–5). ***p < 0.001. One-Way ANOVA *t*-test. (**B**) The percentage of tumor-infiltrating CD86^Hi^ CD11c^+^ DCs in resected tumors was analyzed by flow cytometry (*n* = 3–5). *p < 0.05, **p < 0.01, and ***p < 0.001. One-Way ANOVA *t*-test. (**C**) The density of tumor-infiltrating PD-L1^Hi^ CD11c^+^ DCs in resected tumors was analyzed by flow cytometry (*n* = 3–5). *p < 0.05 and **p < 0.01. One-Way ANOVA *t*-test. (**D**) The density of tumor-infiltrating CD4^+^ T cells in resected tumors was analyzed by flow cytometry (*n* = 3–5). *p < 0.05 and **p < 0.01. One-Way ANOVA *t*-test. (**E**) The density of tumor-infiltrating CD8^+^ T cells in resected tumors was analyzed by flow cytometry (*n* = 3–5). **p < 0.01. One-Way ANOVA *t*-test. (**F**) The density of tumor-infiltrating effector/memory CD8^+^ T_EM_ cells (CD44^+^CD62L^-^CD8^+^ T) in resected tumors was analyzed by flow cytometry (*n* = 3–5). *p < 0.05, **p < 0.01, and ***p < 0.001. One-Way ANOVA *t*-test. (**G**) The density of tumor-infiltrating IFNγ^+^CD8^+^ (IFNγ^+^CD8^+^CD3^+^) T cells in resected tumors was analyzed by flow cytometry (*n* = 3–5). *p < 0.05 and ***p < 0.001. One-Way ANOVA *t*-test. (**H**) The density of tumor-infiltrating IFNγ^+^ NK cells (IFNγ^+^CD49b^+^CD3^+^ NK) in resected tumors was analyzed by flow cytometry (*n* = 3–5). *p < 0.05, **p < 0.01, and ***p < 0.001. One-Way ANOVA *t*-test. (**I**) The density of tumor-infiltrating Foxp3^+^ T_reg_ cells (FoxP3^+^CD25^+^CD4^+^CD3^+^CD45^+^ T). in resected tumors was analyzed by flow cytometry (*n* = 3–5). One-Way ANOVA *t*-test

Additionally, compared with those in the other groups, the mRNA expression of the proinflammatory cytokines *Ifnα2, Ifnβ1*, and *Cxcl10* in the resected tumors was significantly increased in the triple-treatment group, compared to other groups (Fig. 5A and B, and 5C). The recruitment of tumor-infiltrating CD11c^+^ DCs and cytotoxic GzmB^+^ immune cells was also elevated in the triple-treatment group (Fig. 5D and E, and 5F). These results demonstrated that TLC388 can enhance STING signaling to reshape the tumor microenvironment, leading to a greater antitumor immune response. To comprehensively evaluate the immune cell profiles within the tumor microenvironment, we examined the proportions of dendritic cells (MHC^Hi^CD11c^+^ DCs, CD86^Hi^CD11c^+^ DCs and PD-L1^Hi^CD11c^+^ DCs), T cells (CD4^+^ T cells and CD8^+^ T cells), effector/memory T cells (CD44^+^CD62L^−^CD8^+^ T_EM_ cells), cytotoxic T/NK cells (IFNγ^+^CD8^+^ T cells, IFNγ^+^CD49b^+^ NK cells) and regulatory T cells (Foxp3^+^CD25^+^CD4^+^ Treg cells). The gating strategies are shown in Fig. S2. We observed greater infiltration of MHC^Hi^CD11c^+^ DCs and CD86^Hi^CD11c^+^ DCs in the triple-treatment group than in the other groups (Fig. 6A, S3A, 6B, and S3B). The density of PD-L1^Hi^CD11c^+^ DCs was increased by RT plus TLC388, implying that PD-L1 expressed on DCs may diminish T cell–mediated cytotoxicity by attenuating T cell activation via an impaired antigen-presenting ability [43–46] (Fig. 6C and S3C). However, there was no significant increase in the triple-treatment group (Fig. 6C). The density of total tumor-infiltrating CD4^+^ and CD8^+^ T cells was also increased in the triple-treatment group (Fig. 6D and E, and S4A). But the increase in expression was not statistically significant compared to that in the other dual treatment groups (Fig. 6D and E, and S4A). Notably, compared with those in the other groups, the densities of effector/memory CD44^+^CD62L^−^CD8^+^ T_EM_ cells, cytotoxic IFNγ^+^CD8^+^ T cells, and cytotoxic IFNγ^+^CD49b^+^ NK cells were markedly increased in the triple-treatment group, compared to other groups (Fig. 6F, S4B, 6G, and 6H). The percentage of immunosuppressive Foxp3^+^CD25^+^CD4^+^ Treg cells did not increase in the triple-treatment group (Fig. 6I). Taken together, these results demonstrated that TLC388 significantly reinvigorated cancer immunogenicity, enhancing the recruitment of DCs and functional cytotoxic T cells, thereby improving therapeutic efficacy in poorly immunogenic MSS-CRC.

## Discussion

The impact of cytotoxic chemotherapeutics on immune cells is becoming increasingly evident, exhibiting effects that can either suppress or stimulate immune system [47]. Most studies on the immunomodulatory effects of chemotherapeutics on tumor-infiltrating immune cells have primarily focused on overcoming tumor immune escape through the depletion of immunosuppressive cells [48–51]. However, recent findings have indicated that specific subsets of chemotherapeutics can enhance host defense against tumors by generating ‘eat-me’ signals derived from tumor cells, which led to the recruitment of immune cells, ultimately resulting in the elimination of tumors [52, 53]. In this study, we unveiled the potential therapeutic impact of TLC388 in reshaping cancer immunogenicity by triggering cancer intrinsic STING activation for antitumor immunity. TLC388 boosts STING-mediated IFN-I production, revitalizing cancer immunogenicity for processes such as dendritic cell maturation and tumor-specific T-cell activation. Moreover, TLC388 significantly enhanced the therapeutic efficacy of radiotherapy and ICIs in poorly immunogenic MSS-CRC model. Collectively, these results highlight that the novel topoisomerase I inhibitor TLC388 promotes antitumor immunity via STING, offering novel therapeutic avenues to enhance treatment response in ICI-unresponsive tumors, such as MSS-CRC.

The influence of TOP1 inhibitors on antitumor immunity has been less explored compared to other chemotherapeutics, warranting further investigation. In vitro culture experiments suggest TOP1 inhibitors represent a distinct subset of immune-modifying chemotherapeutic agents. Treatment of immature human monocyte-derived dendritic cell (moDCs) with topotecan demonstrated an impact on regulation of maturation-related phenotypes, potentially mediated through the modulation of NF-κB signaling pathways [54]. Kitai et al. demonstrated that topoisomerase I inhibitor topotecan triggers the secretion of DAMPs, promoting DC maturation and activating CD8^+^ T cell, thus delaying tumor growth in vivo [11]. Treatment with topotecan resulted in the secretion of exosomes containing immunostimulatory DNA from cancer cells, further activating DC cell via STING-dependent pathway [11]. Additionally, Lee et al. indicated that topoisomerase I inhibitors generate monocyte-derived antigen-presenting cells by CSF1/CSF1R signaling pathway [8]. These results collectively suggest that topoisomerase I inhibitor has potential to act as an adjuvant, eliciting antitumor immunity by modulating cancer immunogenicity. Consistent with previous studies, our results reveal that the novel topoisomerase I inhibitor TLC388 induced the STING activation, leading to the production of IFN-I. This process enhances cancer immunogenicity, particularly in the context of poorly immunogenic MSS-CRC, thereby reshaping the tumor microenvironment. Our results also demonstrated that TLC388 enriches intratumoral MHCII^Hi^CD11c^+^DCs and CD86^Hi^CD11c^+^DC, capable of proliferation and activation of CD8^+^ T cells, which firstly represents the distinctive immunomodulatory effects of TLC388.

Our previous works have demonstrated a significant association between the release of HMGB1, infiltration of CD8^+^ TILs, and a favorable survival outcome in CRC patients particularly in patients who received neoadjuvant chemoradiotherapy regimen [29, 33, 55]. Although the comprehensive molecular mechanisms underlying TLC388-inducd antitumor immunity remain undetermined, we proposed that TLC388 promotes DC maturation by enhancing tumor antigen presentation through STING signaling and inducing immunogenic cell death such as HMGB1, ANXA1 and CRT [22]. In our previous study, we found a notable release of HMGB1 and ANXA1 under low dose of TLC388, prompting ICD and enhancing cancer immunogenicity in vitro and in vivo. These findings suggest that TOP1 inhibitors may hold the potential to induce ICD and antitumor immunity. In agreement with our observation, McKenzie et al. recently indicated that TOP1 inhibitors enhance the antitumor efficacy of T-cell-based cancer immunotherapy. This suggests that TOP1 inhibitor may contribute the release of tumor antigen, thereby increasing cancer immunogenicity and augmenting the efficacy of immunotherapy [8, 9]. Moreover, TLC388 also upregulated antigen processing proteins such as TAP1 and MHC class I expression, enhancing tumor antigen presentation and attracting infiltration of cytotoxic T lymphocyte [22]. In line with these observations, our current study reveals that TLC388 also induces the phosphorylation of STING, leading for the production of IFN-I. This amplified cancer immunogenicity facilitates dendritic cell maturation and T-cell recognition. Furthermore, a significant delay in tumor growth is observed in combination with radiotherapy and ICIs, accompanied by the recruitment of more tumor-infiltrating cytotoxic T lymphocyte for complete response. These results suggest promising therapeutic strategies to enhance therapeutic efficacy of ICIs in poorly immunogenic tumors. Nevertheless, further studies are needed to optimize dosing and timing of TLC388 administration.

The immunomodulatory effect of the novel TOP1 inhibitor, as demonstrated in this study, hold promise for translation into clinical practice by providing a rationale for chemoimmunotherapy. Our current findings suggest that TOP1 inhibitors represent attractive candidates capable of enhancing the efficacy of cancer immunotherapy. Nevertheless, further studies, including investigations into long-term memory immunity and assessment of dose-limiting toxicities, must be conducted before considering their incorporation to improve the efficacy of immunotherapy in clinical settings. In conclusion, we posit that the novel TOP1 inhibitor TLC388 unveiled may serve as a bridge for the evidence-based combination of chemotherapy and immunotherapy.

### Electronic supplementary material

Below is the link to the electronic supplementary material.

Supplementary Material 1

## Data Availability

No datasets were generated or analysed during the current study.
