# Supplementary material for: Activation of STING by the novel liposomal TLC388 enhances the therapeutic response to anti-PD-1 antibodies in combination with radiotherapy
Source: Cancer Immunol Immunother. 2024 Apr 2;73(5):92. doi: 10.1007/s00262-024-03692-8 (PMC10987363; doi:10.1007/s00262-024-03692-8)
Supplement: Supplementary file 1 — Supplementary Material 1 [file 262_2024_3692_MOESM1_ESM.docx]

**Supporting information**

**Activation of STING by the Novel Liposomal TLC388 Enhances the Therapeutic Response to Anti-PD-1 Antibodies in Combination with Radiotherapy**

**Table S1. Clinicopathological parameters of colon carcinoma patients who received adjuvant chemotherapy (n=115).**

| **Clinicopathological parameters** | **Total no.** | **Tumor STING** | | ***p* value** | |  |
| --- | --- | --- | --- | --- | --- | --- |
|  |  | **High** | **Low** | |  | |
|  | 115 | 42 | 73 | |  | |
| Gender |  |  |  | | 0.71 | |
| Female | 44 | 17 (40.5%) | 27 (37%) | |  | |
| Male | 71 | 25 (59.5%) | 46 (63%) | |  | |
| Age |  |  |  | | 0.21 | |
| <65 | 46 | 20 (47.6%) | 26 (35.6%) | |  | |
| ≥65 | 69 | 22 (52.4%) | 47 (64.4%) | |  | |
| pT stage |  |  |  | | 0.61 | |
| pT1-2 | 104 | 39 (37.5%) | 65 (62.5%) | |  | |
| pT3-4 | 4 | 2 (50%) | 2 (50%) | |  | |
| Undefined | 7 | 5(71.4%) | 2 (28.6%) | |  | |
| Tumor differentiation |  |  |  | | 0.30 | |
| Well to moderate | 105 | 39 (90.7%) | 66(91.7%) | |  | |
| Poor | 3 | 2 (4.6%) | 1 (1.4%) | |  | |
| Unknown | 7 | 2(4.7%) | 5(6.9%) | |  | |
| Lymphovascular invasion |  |  |  | | 0.996 | |
| Absent | 86 | 31 (73.8%) | 55 (75.3%) | |  | |
| Present | 25 | 9 (21.4%) | 16 (21.9%) | |  | |
| Unknown | 4 | 2 (4.8%) | 2 (2.7%) | |  | |
| Perineural invasion |  |  |  | | 0.97 | |
| Absent | 85 | 31 (73.8%) | 54 (74%) | |  | |
| Present | 26 | 9 (21.4%) | 17 (23.3%) | |  | |
| Unknown | 4 | 2 (4.8%) | 2 (2.7%) | |  | |
| MMR status |  |  |  | | 0.5 | |
| MMR-proficient | 35 | 24 (43.6%) | 11 (18.3%) | |  | |
| MMR-deficient | 1 | 1 (1.8%) | 0 (0%) | |  | |
| Unknown | 79 | 30 (54.5%) | 49 (81.7%) | |  | |


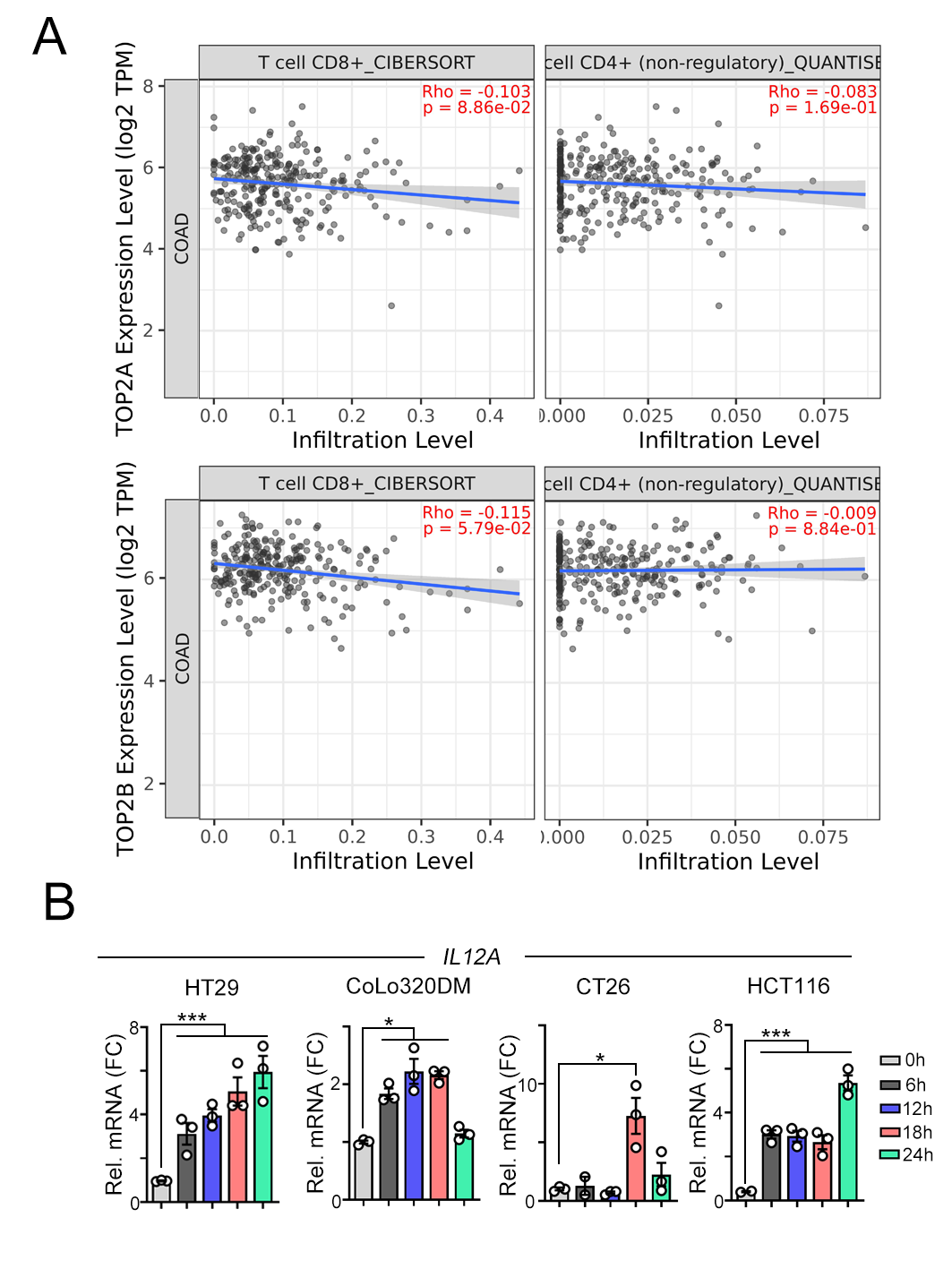

**Figure S1. There is no significant correlation between *TOP2A/TOP2B* mRNA and T cell signatures.**

1. The relationship between *TOP2A/TOP2B* mRNA and T cell signatures.
2. HT29, CoLo320DM, CT26 and HCT116 cells were treated with TLC388 (0.5μM) at various time points (hours). The mRNA level of *IL12A* was analyzed by qRT-PCR (*n*=3). **p*<0.05 and ***p*<0.01. One-Way ANOVA t-test.


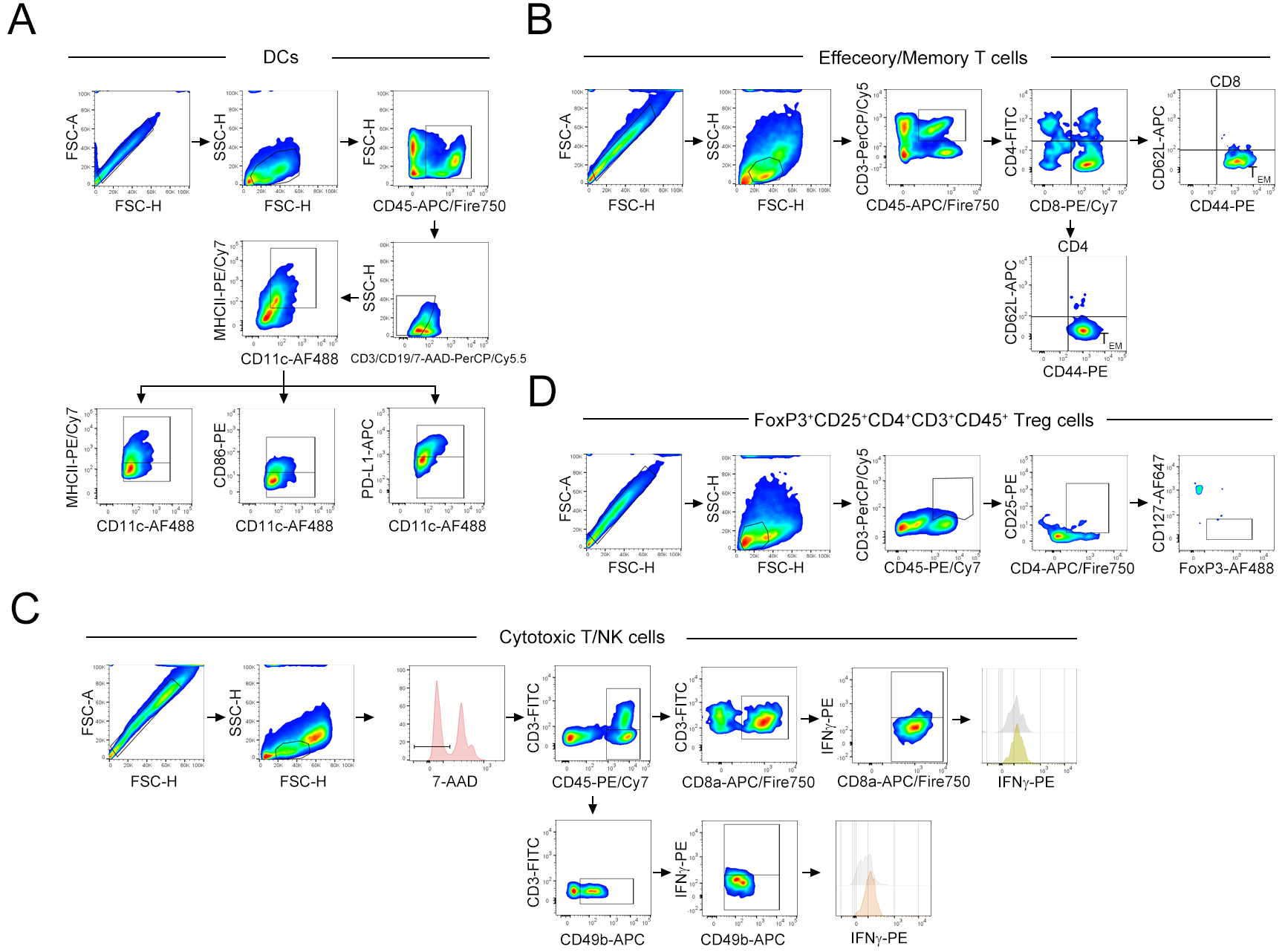

**Figure S2. The gating strategies of immune cell profiles within resected tumors.**

1. The gating strategy for dendritic cells.
2. The gating strategy for CD4 and CD8 T cells.
3. The gating strategy for IFNγ^+^CD8^+^CD3^+^ T and IFNγ^+^CD49b^+^CD3^+^ cells.
4. The gating strategy for FoxP3^+^CD25^+^CD4^+^CD3^+^CD45^+^ Treg cells.


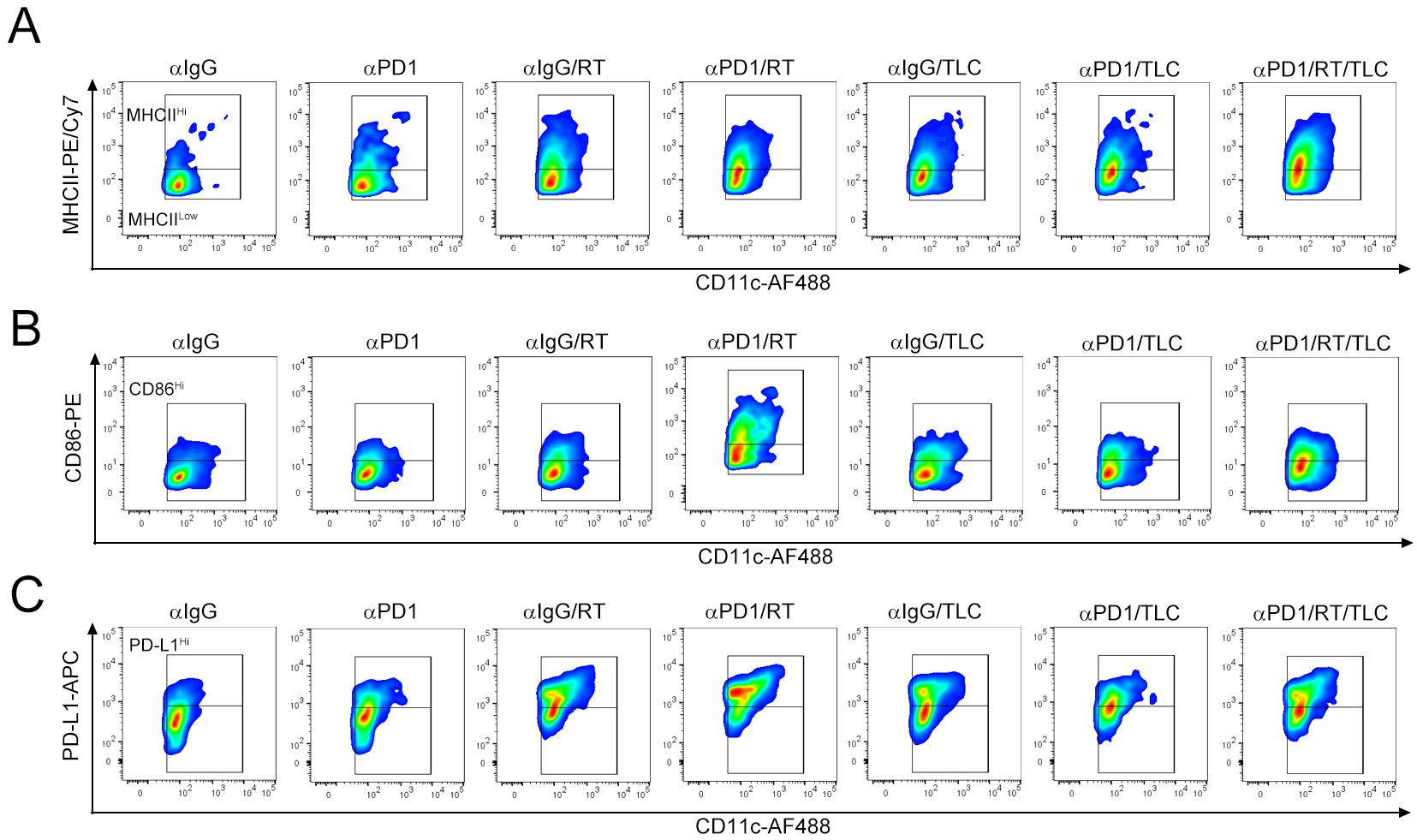

**Figure S3. Combinational therapies significantly reshaped the tumor microenvironment, leading to the recruitment of dendritic cells.**

1. The representative images of tumor-infiltrating MHC-II^Hi^ CD11c^+^ DCs.
2. The representative images of tumor-infiltrating CD86^Hi^ CD11c^+^ DCs.
3. The representative images of tumor-infiltrating PD-L1^Hi^ CD11c^+^ DCs.

**
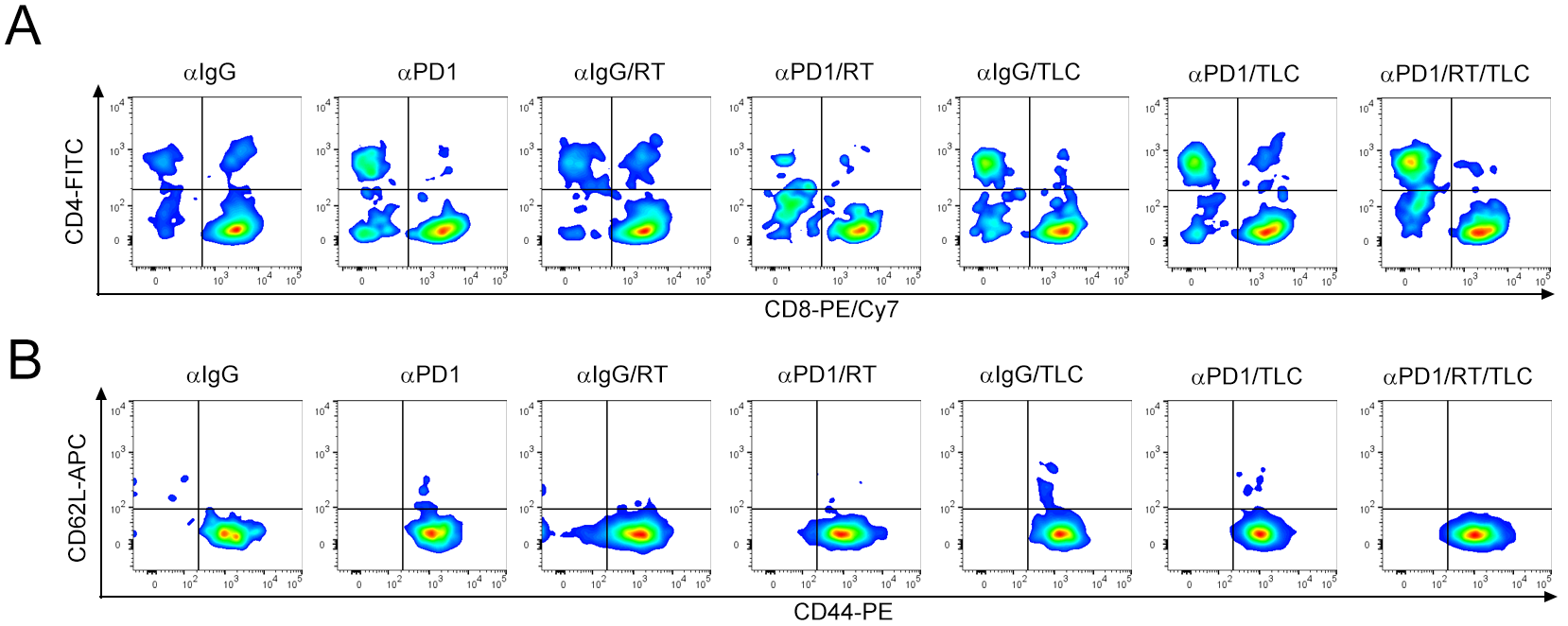
**
**Fig. S4. Combinational therapies significantly reinvigorated the tumor microenvironment, leading to the recruitment of T cells.**

1. The representative images of tumor-infiltrating CD4^+^ T cell and CD8^+^ T cells.
2. The representative images of tumor-infiltrating effector/memory CD44^+^CD62L^-^CD8^+^ T_EM_.
